# Altered volatile emissions and incomplete photosynthetic recovery after heatwave-associated drought and rewetting in the woolly feather-moss *Tomentypnum nitens*

**DOI:** 10.1093/aobpla/plag054

**Published:** 2026-10-15

**Authors:** Annika Engroff, Marta Contreras-Serrano, Jing Tang, Riikka Rinnan

**Affiliations:** Department of Biology, Center for Volatile Interactions (VOLT), University of Copenhagen, Copenhagen 2100, Denmark; Department of Biology, Center for Volatile Interactions (VOLT), University of Copenhagen, Copenhagen 2100, Denmark; Department of Biology, Center for Volatile Interactions (VOLT), University of Copenhagen, Copenhagen 2100, Denmark; Department of Biology, Center for Volatile Interactions (VOLT), University of Copenhagen, Copenhagen 2100, Denmark

**Keywords:** bryophytes, drought, volatile organic compounds, stress, water relations, photosynthesis, global change biology, ecology

## Abstract

Mosses dominate ground cover in many ecosystems and are central to carbon and nutrient cycling, water regulation, and surface energy balance. Their poikilohydric physiology, without stomatal control or vascular transport, couples metabolism closely to moisture. While climate extremes affecting moisture become more frequent, how drought and rewetting affect volatile organic compound (VOC) emissions from mosses remains poorly understood. This study examined how a heatwave-associated drought and subsequent rewetting affected VOC emission rates and blend compositions in the moss *Tomentypnum nitens*. Subarctic heath mesocosms containing *T. nitens* were maintained in climate chambers during a pre drought period and exposed sequentially to a 3-week heatwave-associated drought, and 1 week rewetting treatment. Moss shoots detached from mesocosms were enclosed in a gas-exchange cuvette to measure VOC emissions analysed by GC-MS, net photosynthesis, and evaporation rates. Tissue water content was also determined. Drought strongly reduced water content and evaporation and shifted net photosynthesis from positive to negative values. Total VOC emissions declined by 90% in response to drought and recovered after rewetting (*P* = .06). Drought also shifted the VOC blend towards hydrocarbon dominance (nearly 80% of total). After rewetting, the blend contained a greater proportion of oxygenated VOCs and a smaller proportion of sesquiterpenes than before drought. The legacy effects of drought on VOC blend may influence both the ecological roles of moss VOCs and their contribution to biosphere–atmosphere interactions.

## Teaser

Mosses are often overlooked despite playing an important role in ecosystems. We investigated how drought following a short heatwave, and subsequent rewetting, affected volatile emissions and photosynthesis of the moss *Tomentypnum nitens*. Drought greatly reduced volatile emissions, altered the mixture of compounds released, and shifted net photosynthesis to negative values. After rewetting, net photosynthesis increased again and the total quantity of volatile emissions returned to approximately their previous levels, but the mixture of compounds remained altered. Our findings show that even though mosses in general tolerate changes in humidity, restoring water availability does not necessarily mean that moss fully returns to their previous state. Understanding these responses is particularly relevant as climate change increases the frequency and intensity of extreme weather events.

## Introduction

Mosses cover an estimated 9.4 × 10^6^ km^2^ of the global land surface and can sustain substantial net primary productivity, reaching up to ∼350 g C m^−2^ in some Arctic systems (Zha and Zhuang 2021, Eldridge *et al.* 2023). By dominating the ground layer in many ecosystems, mosses contribute substantially to carbon, nitrogen, and water cycling, and they provide thermal insulation of soils, tree stems, and permafrost (Lindo and Gonzalez 2010, Turetsky *et al*. 2012). Climate change increases the frequency and intensity of heatwaves and droughts (IPCC 2023), exposing moss communities not only to reduced water availability but also to compound or sequential stress events in which high temperatures can precede or accompany drying. Therefore, it is important to assess how mosses respond to and recover from drought in terms of photosynthesis and emissions of volatile compounds as these responses ultimately influence ecosystem functioning.

Mosses are poikilohydric organisms, meaning that their physiological functioning is tightly controlled by tissue hydration and external water availability (Glime 2017a). Because they lack true roots and vascular tissue, and because their leaf surfaces lack stomatal regulation, moss water content rapidly equilibrates with surrounding environmental conditions (Oliver *et al.* 2005, Goetz and Price 2015, Glime 2017a). Thus, water vapour loss from moss shoots is largely governed by evaporation, making evaporation rate a useful measure of moss drying dynamics (Goetz and Price 2015). Photosynthesis often declines rapidly as tissues desiccate, while recovery after rewetting varies among species and depends on drought duration (Schipperges and Rydin 1998, Proctor 2000, Proctor *et al.* 2007a). Although many bryophytes are desiccation tolerant, prolonged drought can result in delayed or incomplete recovery of photosynthetic function (Proctor 2001, Oliver *et al.* 2005, Proctor *et al.* 2007a, 2007b). In this context, tissue water content, evaporative water loss, and net photosynthesis therefore provide complementary measures of hydration, drying and carbon assimilation during drought and recovery.

These physiological responses may, in turn, alter volatile organic compound (VOC) emissions. VOCs are carbon-based compounds that readily enter the gas phase under ambient conditions and include diverse metabolites, such as terpenoids and so-called green leaf volatiles (GLVs), that are produced both constitutively and in response to abiotic and biotic stresses (Bergman *et al.* 2025). Plant VOC emissions reflect both metabolic production and physicochemical release from tissues and may be influenced by photosynthetic carbon supply, enzyme activity, stress responses, and internal storage pools (Niinemets *et al.* 2004, Peñuelas and Staudt 2010). In vascular plants, mild drought often upregulates VOC production, while severe drought suppresses it (Peñuelas and Staudt 2010). Mosses also emit diverse VOCs (Edtbauer *et al.* 2021, Ryde *et al.* 2022, Yáñez-Serrano *et al.* 2024), but we do not know how these emissions are affected by drought. Moss VOCs can originate from multiple metabolic pathways and from intracellular lipids and membranes that may act as internal pools or storage-like reservoirs (Lange 2015, Resemann *et al.* 2019).

Drought may influence VOC production when photosynthetic carbon supply is reduced. Studies from vascular plants have established that recently assimilated carbon is needed for isoprene synthesis (Delwiche and Sharkey 1993, Sharkey *et al.* 2008). Emissions of monoterpenes and sesquiterpenes may likewise draw on recently assimilated carbon (Staudt and Lhoutellier 2011, Ghirardo *et al.* 2020). However, the connection between net photosynthesis and VOC emissions is not universal because VOCs may also originate from storage pools, physicochemical release from tissues or stress related processes associated with membrane disruption (Niinemets *et al.* 2004, Loreto *et al.* 2006, Peñuelas and Staudt 2010). Changes in net photosynthesis and VOC emissions may therefore occur in parallel without necessarily meaning that all VOCs are directly related to photosynthetic rate.

These differences in biosynthetic and physicochemical control also mean that drought may not affect all VOCs proportionally (Niinemets *et al.* 2004, Peñuelas and Staudt 2010). Here, VOC emission rate refers to the absolute quantity emitted per unit drymass and time, whereas VOC blend composition refers to the relative contribution of individual compounds or compound groups to total emissions. Drought and rewetting may therefore alter the total emission rate of all VOCs summed, group specific emission rates and blend compositions in different ways.

Evidence for such responses in mosses remains sparse. In *Sphagnum* moss dominated peat mesocosms, drought reduced overall VOC emissions, induced GLV emissions and caused persistent reduction in sesquiterpene emissions lasting still after rewetting (Männistö *et al.* 2024). This may be because terpenoid emissions are likely related to current carbon supply and enzyme activity, whereas GLVs may arise from non-enzymatic stress responses to cellular damage (Loreto *et al.* 2006), so that VOC recovery after rewetting may be decoupled from the recovery of tissue hydration or photosynthetic activity. Whether these responses apply to other moss species remains unknown.

Here, we assessed how a simulated heatwave-associated drought and subsequent rewetting affect VOC emission rates and VOC blend compositions in the woolly feather moss *Tomentypnum nitens*, and used concurrent measurements of water content, evaporation, and net photosynthesis rate to support the interpretation of the VOC responses. *T. nitens* was used as a model species, as it is a widespread and abundant moss across Northern Hemisphere ecosystems (Turetsky *et al.* 2012, Hedenäs *et al.* 2020, Baisheva 2022). *T. nitens* grown in intact subarctic heath mesocosms was exposed to a controlled climate chamber experiment comprising three sequential treatment periods. These were a well-watered pre drought treatment, a 2-day heatwave at +8°C above baseline followed by a 3-week drought at baseline temperature, and 1 week of rewetting. Independent mesocosms were sampled before the heatwave-associated drought, at the end of drought and after rewetting. Quantifying all response variables simultaneously at these three sampling points allowed to assess whether VOC emissions responded comparably with hydration and carbon assimilation. We hypothesized that (i) heatwave-associated drought would reduce net photosynthesis and the emission rates of total VOCs and VOCs associated with active biosynthesis and recent photosynthetic carbon supply, such as terpenoids; (ii) drought would shift the VOC blend away from its pre drought composition, revealing a higher contribution of stress-related VOCs or compounds less related to active photosynthesis; and (iii) rewetting would increase net photosynthesis and VOC emission rates, but the VOC blend would remain altered due to delayed metabolic recovery or rewetting related stress responses.

## Material and methods

### Collection of moss

The moss *T. nitens* was collected from a subarctic heath in Abisko, northern Sweden near the lake Vassijaure (68°25′41.84″ N, 018°15′ 37.77″ E, 385 m) in September 2023. The moss grew among prostrate and low vegetation, evergreen, and deciduous shrubs (*Empetrum hermaphroditum*, *Vaccinium myrtillus*, *Betula nana*, *Vaccinium vitis-idaea*, and other graminoids). Blocks of this vegetation and the soil beneath were sampled and placed into plastic containers as mesocosms (dimensions for the soil within the plastic containers were 19 cm × 19 cm × 10 cm, L × W × H; Contreras-Serrano *et al.* 2025). In total, 13 mesocosms were collected and used to grow the mosses within the vegetation community.

### Experimental set up

The experiment builds on the climate chamber study conducted by Contreras-Serrano *et al.* (2025). The climate chambers were programmed to maintain the same baseline conditions according to long-term climate data from Abisko over the past 30 years (Swedish Meteorological and Hydrological Institute 2022). Climate simulations (Fig. 1a) began after the mesocosms were transported ex-situ and placed into climate chambers (FitoClima 1200, Aralab, Rio de Mouro, Portugal). Autumn was simulated for 28 days at 5–10°C, 70%–85% relative humidity (RH) and maximum photosynthetic photon flux density (PPFD) of 200–400 μmol m^−2^ s. Winter was then simulated for 81 days to induce plant dormancy under darkness, 2–5°C and 70%–75% RH. Spring simulations were initiated after winter which lasted 46–50 days at 6–11°C, 60%–80% RH and 840 μmol m^−2^ s PPFD. This was followed by the summer simulation which marked the start of the experiment. During summer, the mesocosms were kept at a minimum 9°C night temperature and maximum 14°C day temperature. RH was adjusted according to temperature ranging from 72% to 82%. A 17 h daylight cycle was maintained with 611 µmol m^−2^ s^−1^ PPFD. CO_2_ concentration was held constant across treatments and seasons at 420 ppm. Mesocosms were irrigated and surface sprayed with double demineralized water twice a week to keep relative soil water content (RSWC) at approximately 50%–60% and the moss moist (Contreras-Serrano *et al.* 2025).

**Figure 1:**
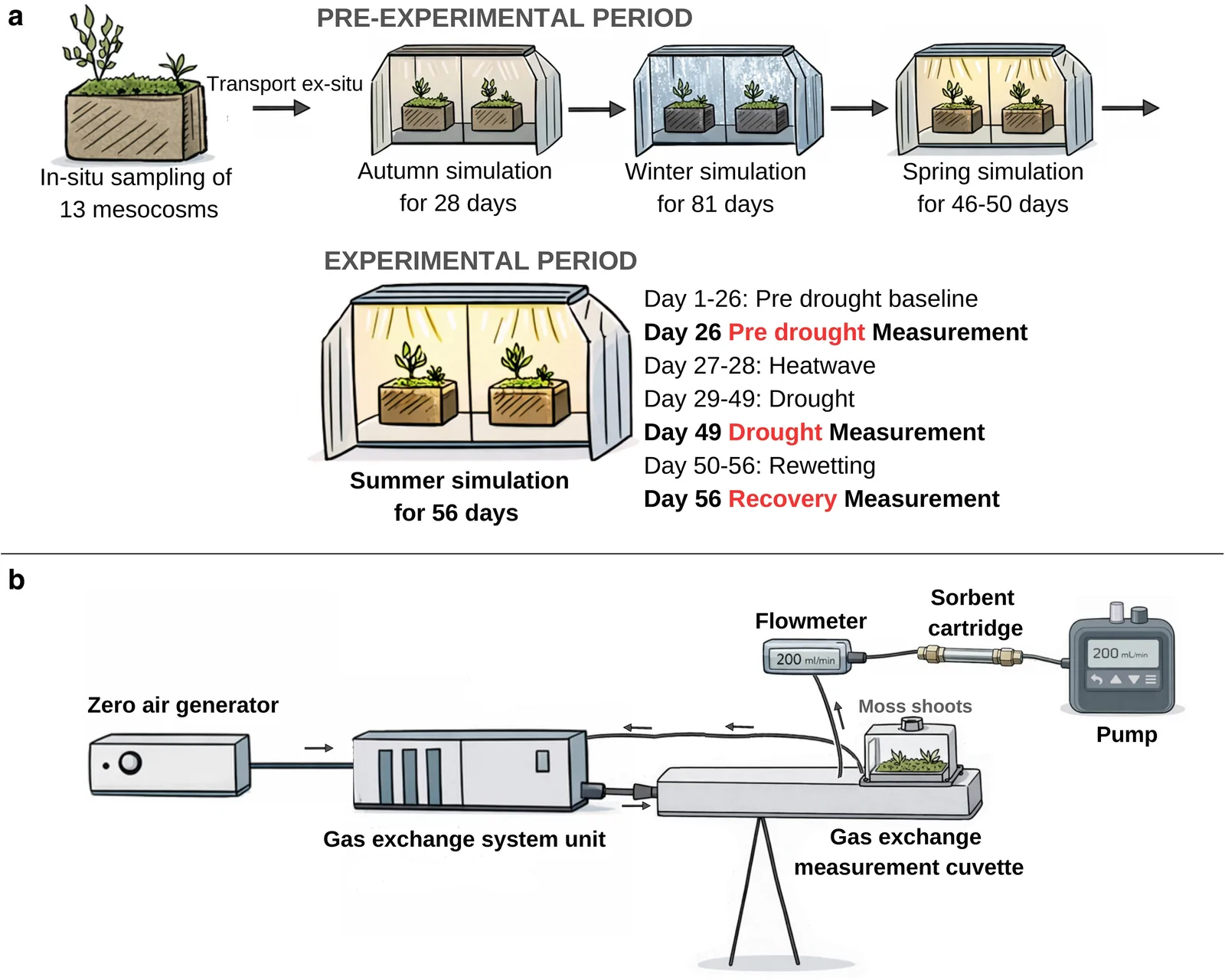
Schematic of (a) the experimental timeline and measurement time points and (b) the set up to simultaneously measure net photosynthesis rate and VOC emissions via the gas exchange system, inspired by Contreras-Serrano *et al*. (2025). (a) Mesocosms were collected from a Subarctic heath, transported to the laboratory, and placed in climate chambers where they underwent a pre-experimental period of simulated seasons before the actual experiment run under summer conditions. Pre-drought measurements were taken after the first 26 days of summer. A 2-day heatwave of +8°C was then applied. Summer temperature was then restored and watering withheld for the duration of a 3-week drought. Drought measurements were taken at the end of this period. Watering was then resumed, and recovery measurements were taken after 6 days. (b) A gas exchange system was used to measure net photosynthesis. Purified air from a zero-air generator was used as the inlet air to the cuvette where the moss sample was enclosed. A subset of the outflow from the cuvette was drawn through a sorbent cartridge using a pump for VOC analysis.

After 26 days under summer conditions, a 3-week drought treatment was initiated by withholding water, with a 2-day heatwave (+8°C above baseline; 51%–61% RH) imposed at its onset before temperature was returned to baseline summer conditions. The heatwave temperature represented the largest mean anomaly among Abisko heatwave events identified from historical (1984–2014) and projected (2015–2100) daily mean temperatures, with heatwaves defined as at least three consecutive days above the 95th percentile (Swart *et al.* 2019, Contreras-Serrano *et al.* 2025). The drought duration was based on a pilot test showing that a 3-week period was sufficient to reduce RSWC below 20%. Following the drought treatment, the remaining mesocosms were rewetted to 50%–60% RSWC and watered and surface-sprayed once again every 2 days during the 1-week recovery treatment (Contreras-Serrano *et al.* 2025).

The experiment comprised 13 mesocosms, each providing one independent *T. nitens* sample. Mesocosms were randomly assigned to one of three sampling stages and sampled only once at pre drought (*n* = 4), at the end of the 3-week drought treatment (*n* = 5) or at the end of the 1-week recovery treatment (*n* = 4). Each mesocosm was discarded after its sample was collected and measured.

### Gas exchange and VOC measurements

A sample of 9–14 active, green *T. nitens* shoots (each ca. 4 cm long) were carefully detached from each mesocosm for the measurements and pooled to form one sample. Shortly thereafter, the sample was arranged in a single layer in a 3.2 × 4 cm cuvette (Lichens/Mosses Cuvette 3010-V3) and measured using the GFS-3000 portable photosynthesis system (Walz, Effeltrich, Germany; Fig. 1b). The instrumentation for gas exchange and VOC measurements was placed inside a climate chamber held at the same baseline settings as the experiment. During the measurements of the moss samples, the climate chamber and cuvette set-points were kept steady at 14°C and RH of 74%. Once a stable gas exchange rate was observed, exchange of CO_2_ and water was logged every 5 min for 20 min of which the final 5–15 min was averaged to yield net photosynthesis and evaporation rate. A cuvette flow with a constant airflow of 750 µmol s^−1^ of zero air was provided by ultra-pure air generator (747-30 Advanced Ultra-Pure Air Generator; Tisch Environmental, Cleves, OH, USA). Simultaneously, a subsample of the cuvette headspace was drawn for VOC analysis through a stainless-steel sorbent cartridge containing Tenax TA and Carbograph 1TD sorbents (Markes International, Llantrisant, UK). VOC sampling was done at a flow rate of 200 ml min^−1^ using a pump (AirCheck TOUCH; SKC Ltd., Blandford Forum, UK). Each sampling day, one blank was obtained following the same procedure as above but without any moss sample in the cuvette. The blank was later subtracted from the samples before calculating VOC emission rates.

The measurement cuvette was carefully cleaned using ethanol on a cotton swab between sample days and left open to ensure no trace VOCs in the cuvette. Before VOC sampling, the fresh weight (FW) of the samples was determined and after sampling, each moss sample was oven-dried at 70°C for 72 h and dry weight (DW) was recorded. Moss water content (%) was calculated as (FW-DW)/FW*100. Photosynthesis, evaporation and VOC emission rates were proportioned to sample dry weight.

### Thermal desorption, GC-MS analysis, and calculation of VOC emission rates

Sorbent cartridges containing the sampled VOCs were analysed via GC-MS [7890A Series GC coupled with 5975C inert MSD/DS Performance Turbo EI System (Agilent, Santa Clara, CA, USA)] after thermal desorption in a TD100-xr thermal desorber (Markes International Ltd., Llantrisant, UK). Desorption of the cartridges was done with an oven programme that released VOCs onto a HB-5MS capillary column (50 m length, 200 μm diameter, 0.33 μm film thickness) and a helium flow of 1.2 ml/min was used to transport the sample through the capillary column. The GC oven programme started at 40°C (3 min hold), ramping at a rate of 5°C min^−1^ to 210°C and then at a rate of 20°C min^−1^ to 250°C (8 min hold). The mass spectrometer acquired spectra with an m/z range of 35–300. Chromatograms were analysed using PARADISe v6.0.1 (Johnsen *et al.* 2017). Compounds were identified by comparison to 45 authentic standards (Supplementary Table S1) and tentatively with the NIST23 mass spectral library (National Institute of Standards and Technology, Gaithersburg, MD, USA). Concentrations were quantified using the standard compounds as in Ryde *et al.* (2022). Compounds were categorized into monoterpenes (MTs), sesquiterpenes (SQTs), oxygenated volatile organic compounds (OVOCs), green leaf volatiles (GLV), hydrocarbons (HCs), and all the rest were categorized as OTHER. VOC emission rates were calculated according to the equation for dynamic enclosure measurements (Ortega and Helmig 2008) and normalized to sample dry mass, yielding values in nmol g^−1^ h^−1^.

### Statistical analyses

Statistical analyses were conducted in the R statistical framework version 4.5.0 (R Core Team 2026). Differences among treatments (pre drought, drought, and recovery) in net photosynthesis rate, evaporation rate, water content, and VOC emission rates of *T. nitens* were analysed via linear model fitted with function gls() from the nlme package, allowing for treatments specific residual variances. Because each sample was measured only once and there was no additional grouping structure, it provided a way to handle the unbalanced sample sizes and heteroscedasticity among treatments. When overall treatment effect was significant for analysis of variance (ANOVA), we used Tukey’s test for *post hoc* comparisons of estimated marginal means using the emmeans package, to test which treatment levels differed from another. For net photosynthesis, evaporation rate and water content, assumptions of normality and homogeneity of variances were met as confirmed via Shapiro–Wilk and Levene’s test (*P* > .05) and for VOCs, log-transformations were applied to reach assumptions of ANOVA.

Principal component analysis (PCA) was used to assess whether the treatments separated from each other based on the VOC blends, indicating differences in overall VOC fingerprints. The PCA was done on log-transformed unit variance-scaled and centred data using SIMCA 17 (Sartorius AG, Goettingen, Germany).

## Results

### Effect of drought and rewetting on net photosynthesis, evaporation rate, and moss water content

Net photosynthesis rate of *T. nitens* differed significantly among treatments (Fig. 2a; ANOVA, *P* = .001). Pre drought, *T. nitens* showed positive net photosynthesis rates which decreased by 164% in response to drought, turning negative. Rewetting recovered net photosynthesis rates to ∼17% of the pre drought mean rate resulting in a slightly positive net rate, which was not significantly different from pre drought or drought treatments.

**Figure 2:**
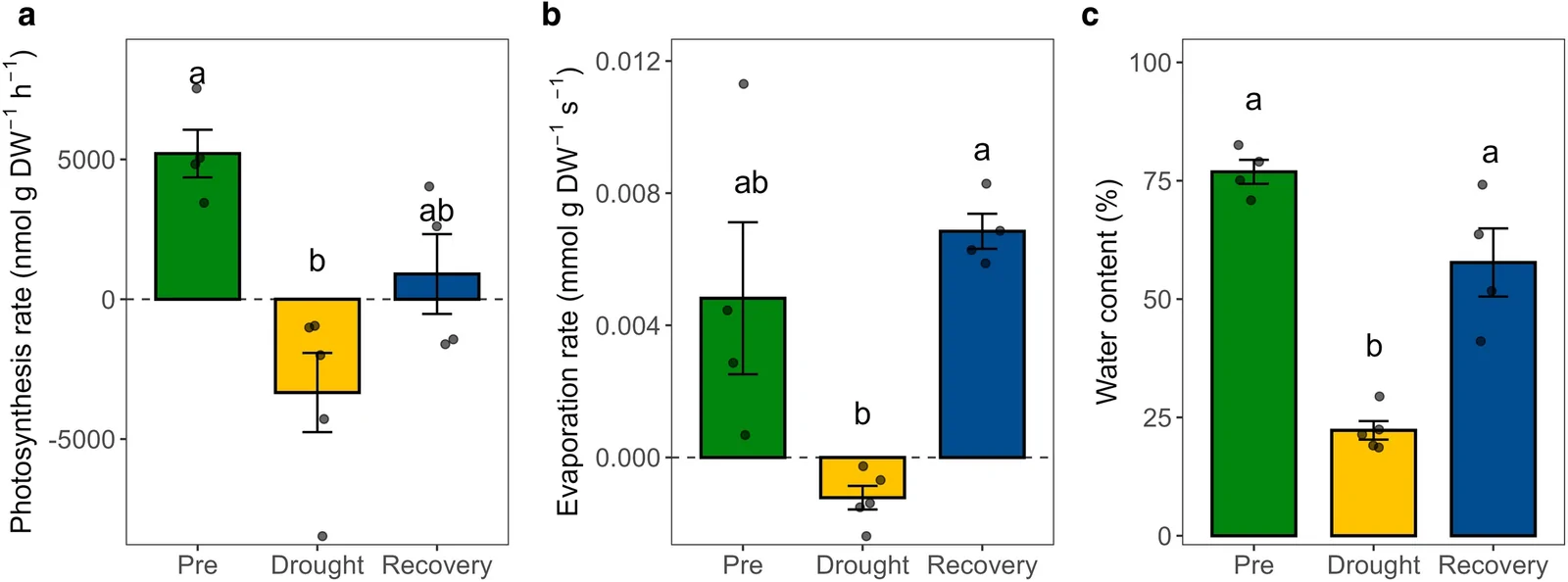
Average net photosynthesis rate (a), evaporation rate (b) and water content (c) ± standard error (*n* = 4–5), measured in *T. nitens* in pre drought, drought, and recovery treatments. Points show the values of each individual sample. The bars that share the same letter do not differ significantly from each other (*P* < .05, Tukey’s test).

At the same time, evaporation rate (Fig. 2b) measured in drought treated moss samples turned from positive to negative, indicating that the moss was so dry that it absorbed water vapour from the air instead of losing water vapour. After rewetting the evaporation rate recovered again.

The water content of *T. nitens* was significantly reduced by the drought treatment (Fig. 2c; ANOVA, *P* < .001). The drought treatment caused a decline in water content by 71% (from 76.9 ± 2.5% to 22.3 ± 1.9%). Water content recovered to 57.7 ± 7.2% after rewetting but was still 25% lower compared to the pre drought water content.

### Effect of drought and rewetting on VOC emission rates

Total VOC emission rates were only marginally affected by treatment (Fig. 3, *P* = .06) but followed the general trend of reduced emission rates in drought treated moss. Total VOC emission rate was substantially suppressed by drought, decreasing by ∼90% relative to pre drought. Following rewetting, total VOC emission rates recovered to approximately pre drought rates, reaching ∼109% of the pre drought mean rate. At the VOC-group level, emission rates generally mirrored this pattern, with several groups showing significant treatment effects (*P* < .05, Fig. 4).

**Figure 3:**
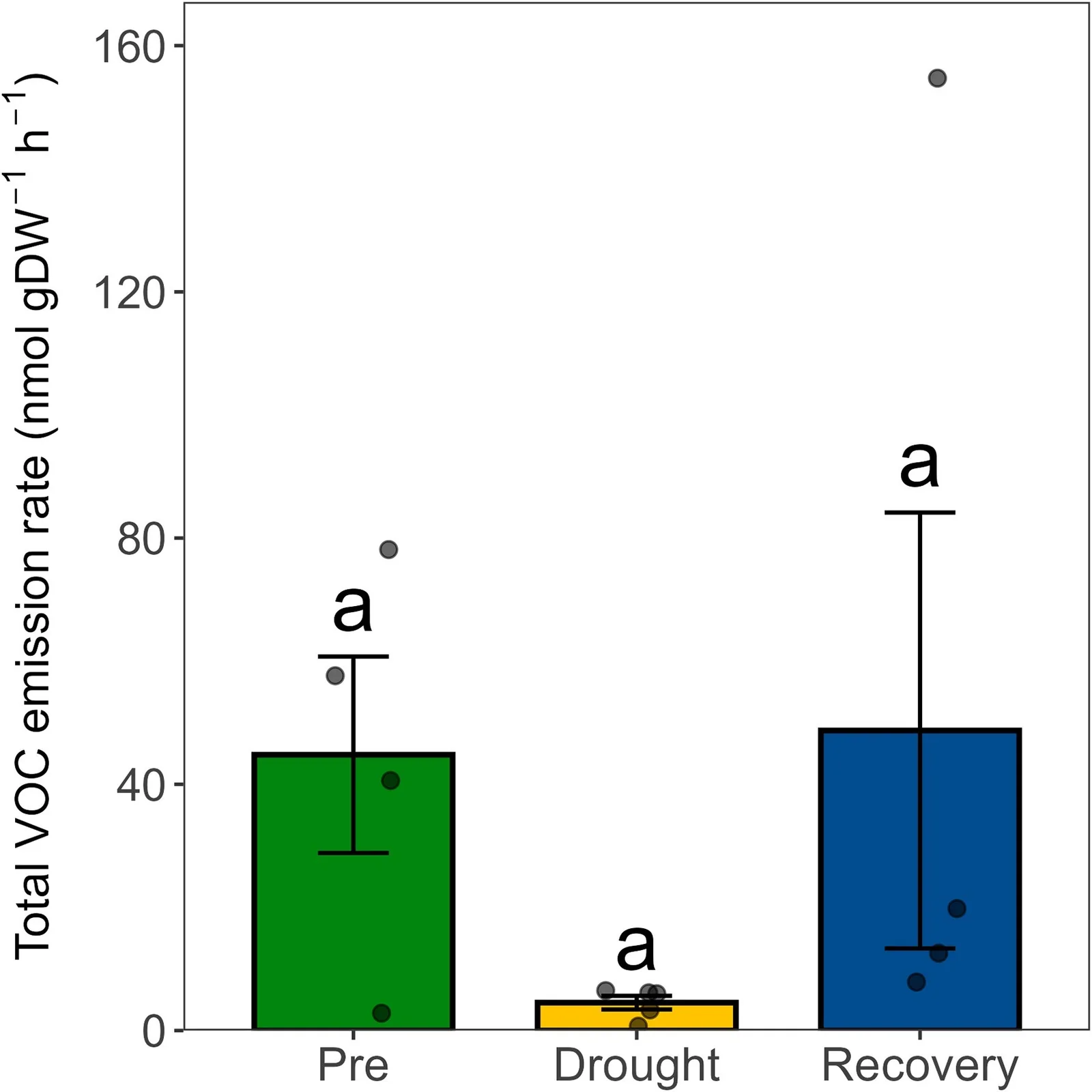
VOC emission rates (nmol g DW^−1^ h^−1^, mean ± standard error) of total VOC emissions measured for *T. nitens* within pre drought, drought, and recovery treatment (*n* = 4–5). Points show the values of each individual sample. The bars that share the same letter do not differ significantly from each other (*P* < .05, Tukey’s test).

**Figure 4:**
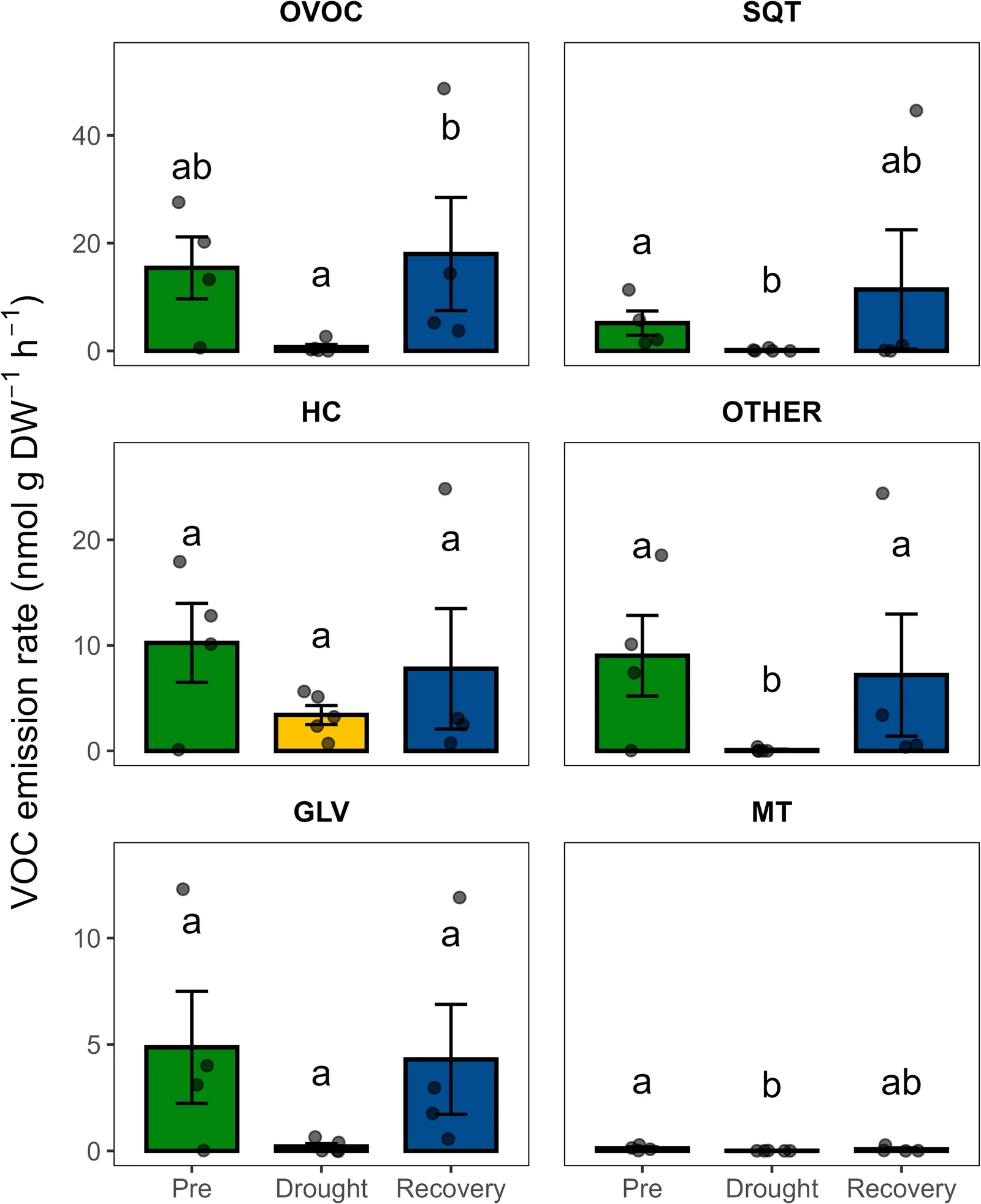
VOC emission rates (nmol g DW^−1^ h^−1^, mean ± standard error) of each VOC group measured for *T. nitens* within pre drought, drought, and recovery treatment (*n* = 4–5). GLV = Green leaf volatiles, HC = Hydrocarbons, MT = Monoterpenes, SQT = Sesquiterpenes, OVOC = oxygenated volatile organic compounds, OTHER = all unknown compounds and compounds not in the beforementioned groups. Points show the values of each individual sample. The bars that share the same letter do not differ significantly from each other (*P* < .05, Tukey’s test).

Across VOC groups (Fig. 4), the drought treatment strongly suppressed emission rates relative to pre drought (reductions of −66.7% to −99.1% across groups). OVOC emission rates declined from 15.4 ± 5.7 to 0.7 ± 0.5 nmol g^−1^ h^−1^ (−95.5%, *P* = .05). SQT emission rates decreased from 5.2 ± 2.3 to 0.15 ± 0.1 (−97.2%, *P* = .03). HC emission rates dropped from 10.2 ± 3.7 to 3.4 ± 0.9 (−66.7%), but showed no overall significant treatment effect (*P* = .90). OTHER compound emission rates declined from 9.0 ± 3.8 to 0.08 ± 0.08 (−99.1%, *P* = .04). GLV emission rates decreased from 4.9 ± 2.6 to 0.2 ± 0.1 (−95.7%); with marginal significance (*P* = .057). MT emission rates declined from 0.1 ± 0.05 to 0.005 ± 0.003 (−96.1%, *P* = .02).

Following rewetting, mean emission rates shifted relative to pre drought values but with no significant differences to pre drought. OVOC emission rates increased to 18.0 ± 10.5 (+16.9% vs pre drought), SQT to 11.4 ± 11.1 (+120.9% vs pre drought), HC emission rates to 7.8 ± 5.7 (−23.6% vs pre drought), OTHER compound emission rates to 7.2 ± 5.8 (−20.4% vs pre drought), GLV emission rates to 4.3 ± 2.6 (−11.5% vs pre drought), and MT emission rates averaged 0.08 ± 0.06 (−40.2% vs pre drought).

### Effect of drought and rewetting on VOC blends

The relative contribution of VOC groups to the total emission rate revealed shifts in blends of the different treatments (Fig. 5a). The pre drought VOC blend was dominated by OVOCs (30.6 ± 3.8%) and SQTs (27.6 ± 16.2%), with HCs contributing 18.5 ± 4.9%, GLVs 7.8 ± 3.3%, OTHER compounds 15.2 ± 4.9%, and MTs remaining minor with 0.3 ± 0.07%. Under drought, HCs became predominant, accounting for 79.1 ± 8.7% of total VOC emissions, while OVOCs declined to 12.1 ± 7.6%, SQTs to 2.7 ± 1.8%, GLVs to 4.6 ± 2.8%, OTHER to 1.3 ± 1.2%, and MTs stayed negligible (0.1 ± 0.05%). Following rewetting, the composition largely recovered, with OVOCs rising above pre drought contributions with 49.9 ± 11.3%. HCs fell to 15.2 ± 2.2%, MTs remained negligible with 0.1 ± 0.04% while SQTs partially recovered to 10.4 ± 6.8% but remaining below pre drought blend contribution. GLVs increased to 11.8 ± 3.9% above pre drought blend contribution, and OTHER returned to 12.5 ± 5.6%. Overall, drought shifted the blend strongly towards HCs, whereas rewetting led to elevated oxygenated VOCs together with only a partial recovery of sesquiterpenes and an increase in GLVs relative to pre drought.

**Figure 5:**
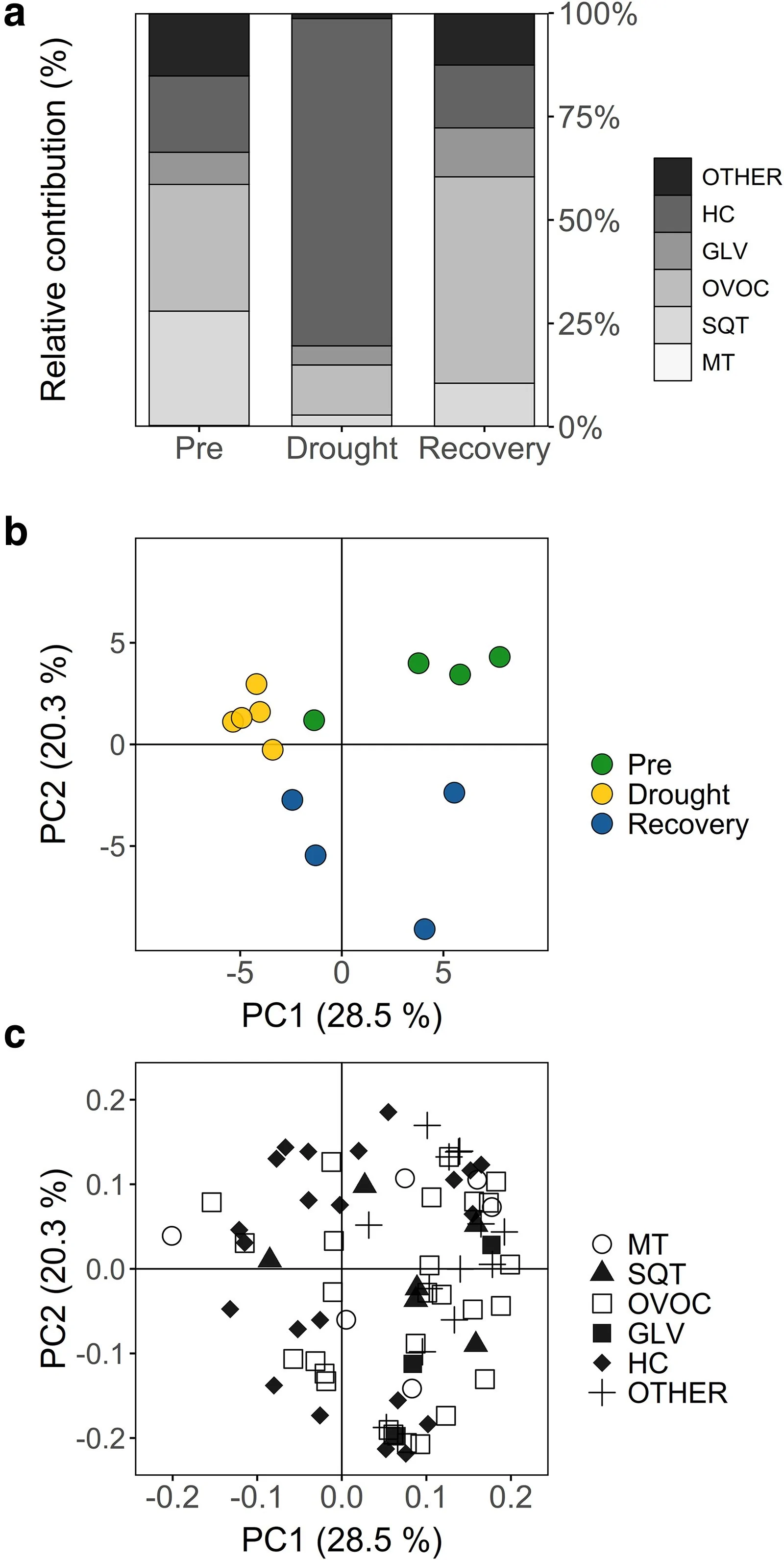
(a) Shows the relative contribution (%) of VOC groups to the total emission rate showing the VOC blend emitted by *T. nitens* in the different treatments (*n* = 4–5). (b) and (c) show the principal component analysis (PCA) of *T. nitens* VOC blend across treatments. (b) represents the score plot of samples (*n* = 4–5 per treatment), showing treatment related differences in VOC blend composition. (c) represents the loading plot showing the contribution of individual VOCs to PC1 and PC2. Symbols denote VOC groups. (MT: monoterpenes, SQT: sesquiterpenes, OVOC: oxygenated VOCs, GLV: green leaf volatiles, HC: hydrocarbons, OTHER: unknown compounds or compounds not belonging to the before-mentioned groups). Percent variance explained by each principal component (PC) is shown in parentheses.

A total of 77 individual VOCs were detected of which 17 were categorized as unknown (Supplementary Table S2). Principal component analysis (PCA) of the VOC blend composition revealed clear treatment-related shifts along the first two axes (Fig. 5b), indicating distinct VOC blends among the pre drought, drought and rewetting treatments. PC1 explained 28.5% of the total variance and primarily captured the contrast between pre drought and drought treatment, with drought samples clustering on the negative side of PC1 and pre drought samples on the positive side. Rewetted samples did not overlap with the pre drought cluster and instead showed greater dispersion, reflecting a more variable blend during recovery. PC2 accounted for a further 20.3% of the variance and separated recovery (negative PC2) from pre drought and drought samples (positive PC2), suggesting that rewetting might have induced a compositional shift that was not simply a return to the pre drought blend.

Loadings indicated that this treatment separation was driven by changes across VOC groups (Fig. 5c; Supplementary Table S2). Compounds with positive PC1 loadings (dominated by OVOCs and including contributions from SQTs and MTs) loaded towards the pre drought samples, whereas compounds with negative PC1 loadings (notably including HCs) aligned with drought samples. Negative PC2 loadings (OVOCs, HCs, and two of three detected GLVs), were consistent with the distinct positioning of rewetted samples along PC2. Together, the PCA indicates that the drought treatment shifted VOC blend, and that rewetting led to a blend that differed from both the drought and pre drought treatment and was more variable among samples.

## Discussion

Heatwaves can precede or accompany drought (Sutanto *et al.* 2025), yet little is known about how such sequential stress and subsequent rewetting affect VOC emissions from widely distributed mosses. This study therefore examined how a 2-day heatwave followed by a 3-week drought and 1 week of rewetting affected VOC emission rates and blend compositions in *T. nitens*, while concurrent measurements of water content, evaporation, and net photosynthesis rate provided context for the physiological state of the moss during VOC sampling. The heatwave-associated drought treatment strongly reduced moss water content and net photosynthesis rate, suppressed VOC emission rates across most compound groups, and shifted the remaining low emissions towards hydrocarbons. After 1 week of rewetting, total VOC emission rates returned to values comparable with pre drought, and water content, evaporation, and net photosynthesis rate increased, whereas the VOC blend composition remained distinct from the pre drought blend. Thus, VOC emission rates and compositions followed different recovery patterns after rewetting.

### Moss water content and gas-exchange responses to heatwave-associated drought and rewetting

The observed 71% decline in water content and the change from positive to negative evaporation rate show that the heatwave-associated drought strongly desiccated *T. nitens*. The negative evaporation rate indicates that the dry moss tissue absorbed water vapour from the humid cuvette air rather than losing water, supporting strong water limitation during VOC measurements. The hydration status of the moss is therefore critical for interpreting VOC emissions, as moss tissues rapidly equilibrate with atmospheric moisture and lose metabolic activity as external water availability declines (Oliver *et al.* 2005, Goetz and Price 2015, Glime 2017b).

Because the drought period was preceded by a short heatwave, the pronounced desiccation likely reflects the combined effects of water limitation and prior heat stress, which cannot be separated in our experiment design. In the desert moss *Syntrichia caninervis*, drying following heat exposure altered water loss, photosynthetic activity, and regenerative capacity (Xu *et al.* 2009). Another field study showed that heatwaves or hot-drought conditions can strongly impair peat moss performance, including causing Sphagnum die-off (Bragazza 2008). Although these studies did not examine VOC emissions from *T. nitens*, they demonstrate that moss performance can be strongly affected when heat exposure and drying are combined or follow one another.

The simultaneous decline of net photosynthesis below zero further indicates that the drought treatment strongly constrained photosynthetic carbon gain. In poikilohydric bryophytes, desiccation causes photosynthetic metabolism to stop quickly, whereas mitochondrial respiration can continue temporarily, leading to net CO_2_ release (Proctor 2000, Proctor *et al.* 2007b). The negative net photosynthesis rate means that, during drought, *T. nitens* had limited supply of recently assimilated carbon for *de novo* VOC biosynthesis.

### Effects of heatwave-associated drought on VOC emission rates and blend composition

The sharp decline in VOC emissions following the heatwave-associated drought treatment supports our first hypothesis that drought would reduce emission rates of total VOCs and VOCs associated with active photosynthesis. Although the decrease in total VOC emissions was only marginally significant statistically due to high variation, the mean magnitude of the decrease was large with total emissions reduced by approximately 90%. Several VOC groups also declined significantly or marginally, including MTs, SQTs, OVOCs, and GLVs. This shows that the drought response was a broad suppression of absolute VOC release across most compound groups.

In pre drought *T. nitens*, the VOC blend was diverse and dominated by OVOCs and SQTs, consistent with the known VOC profile of this species (Ryde *et al.* 2022). Under drought, however, MT and SQT emission rates declined to near-zero values. This response is consistent with the sensitivity of *de novo* terpenoid production to declines in photosynthetic carbon supply and metabolic activity. Emissions of isoprene and several MTs and SQTs have been shown to depend on CO_2_ assimilation and photosynthetic electron transport, linking their biosynthesis to photosynthetic carbon and energy supply (Delwiche and Sharkey 1993, Staudt and Lhoutellier 2011). Reduced supply of carbon precursors, together with drought-induced constraints on enzyme activity, have likely contributed to the decline in MT and SQT emissions (Sharkey *et al.* 2008, Laothawornkitkul *et al.* 2009, Peñuelas and Staudt 2010), although changes in storage pools and diffusion may have additionally contributed. Overall, the responses resemble those observed by Männistö *et al.* (2024) in Sphagnum-dominated mesocosms, where drought reduced total VOC, isoprene and SQT emissions (Männistö *et al.* 2024).

In line with our second hypothesis, heatwave-associated drought altered VOC blend composition, although not through an increase in stress-related GLVs. In the drought treatment, HCs accounted for nearly 80% of total VOC emissions because their absolute emission rates declined less than those of other compound groups. Consequently, the VOC blend shifted from being dominated by OVOCs and SQTs under pre drought conditions to a low-emission blend enriched in HCs. This pattern suggests that drought suppressed emissions that depend strongly on active photosynthesis and *de novo* biosynthesis, while compounds less closely linked to active photosynthesis became relatively more prominent.

The high relative contribution of HCs may reflect both physiological and physicochemical controls. Some HCs may originate from moss lipid pools and therefore depend less on active metabolism than *de novo* produced terpenoids (Niinemets *et al.* 2004, Rissanen *et al.* 2018, Lu *et al.* 2019, Sander 2023). In addition, desiccation can induce oxidative stress and lipid peroxidation, altering membrane and storage lipids and potentially maintaining pools of HCs and other non-oxygenated compounds available for release even when metabolism slows (Guschina *et al.* 2002, Niinemets *et al.* 2004, Proctor *et al.* 2007b, Glime 2017c, Lu *et al.* 2019). Physicochemical factors may further contribute to this response. Because HCs are less water-soluble than OVOCs, they are likely less strongly retained within the external water film surrounding hydrated moss tissues (Sander 2023). As this water film disappears during desiccation, the diffusion barrier for gases is reduced (Cowan *et al.* 1992, Weston *et al.* 2015), so that poorly soluble HCs are released more readily than more water-soluble OVOCs (Niinemets and Reichstein 2003). Together, these mechanisms provide a plausible explanation for the HC-enriched VOC blend observed in response to drought.

### Effects of rewetting on VOC emission rates and blend composition

Consistent with our third hypothesis, 1 week of rewetting restored total VOC emission rates to levels comparable to those measured before drought coinciding with increased tissue water content, positive evaporation rates, and higher net photosynthesis. However, VOC blend composition remained distinct from pre drought measurements.

The recovery blend was characterized by a higher contribution of OVOCs, which accounted for nearly half of total emissions, while SQTs represented a clearly smaller proportion than before drought. Elevated OVOCs may reflect renewed metabolic activity following rewetting, but they can also indicate rehydration stress. Rapid water uptake by previously desiccated tissues can disrupt membranes and trigger oxidative processes, leading to enhanced production of oxygenated compounds. This interpretation is further supported by the stronger association of the GLVs heptanal and hexanal with rewetted samples in the PCA, as these oxygenated compounds are commonly linked to membrane damage, wounding or abiotic and biotic stress responses (Loreto *et al.* 2006, Vicherová *et al.* 2020, Morales-Sánchez *et al.* 2023, Männistö *et al.* 2024).

Although total VOC emissions recovered, the relative contribution of SQTs remained lower than before drought due to elevated OVOC emissions. This contrasts with the results reported by Männistö *et al.* (2024), who found that absolute sesquiterpene emissions from *Sphagnum* peat mesocosms remained significantly reduced for several weeks during post-drought recovery (Männistö *et al.* 2024). Despite major differences between *T. nitens* and *Sphagnum* in cell architecture (single laminal versus large hyaline cells) and thus water conduction traits (Vitt and Hamilton 1975, Hájek and Beckett 2008), both studies suggest that the SQT component of moss VOC blends may remain altered after drought. This may reflect delayed reactivation of terpene biosynthetic pathways or the metabolic costs associated with restoring terpene synthase activity. A complementary perspective is provided by Esteban *et al.* (2024), who found that heptane extracted MT and SQT pools increased with dehydration and remained elevated after rehydration in three bryophyte species. In vascular plants, terpenes originate both from *de novo* biosynthesis and pre-existing storage pools (Lange 2015, Sørensen *et al.* 2020). This may also be relevant for bryophytes, where intracellular lipids and membranes may act as storage-like reservoirs (Lange 2015, Resemann *et al.* 2019, Esteban *et al.* 2024).

These compositional shifts may be ecologically relevant even when total emission rates recover. Vicherová *et al.* (2020) proposed that VOCs released from desiccated and rehydrated mosses can act as warning cues perceived by neighbouring mosses. Although receiver responses were not examined here, the OVOC-enriched and relatively SQT-depleted blend observed after rewetting represents a chemically distinct signal that could influence interactions within moss communities. Future studies should work on (i) separating heatwave and drought effects and applying a range of drought lengths, severities, and repeated dry-rewet cycles and (ii) following recovery across multiple time points.

## Conclusions

This study demonstrates that heatwave-associated drought strongly alters both the magnitude and composition of VOC emissions from *T. nitens*, and that these two aspects of VOC exchange recover differently. The drought treatment caused severe desiccation, reduced net photosynthesis, and suppressed VOC emissions across most compound groups, resulting in a low-emission blend enriched in hydrocarbons. After 1 week of rewetting, total VOC emission rates had recovered to levels comparable with those measured before drought, yet the VOC blend remained distinct from its pre drought state. This decoupling between recovery of emission rates and blend composition suggests that measuring total VOC emissions alone would not fully capture the effects of climatic stress on moss VOC exchange. Even when physiological activity resumes, the chemical information in the VOC blends can retain signatures of previous stress. As droughts and heatwaves are projected to become more frequent, such legacy effects may influence both the ecological roles of moss VOCs and their contribution to ecosystem–atmosphere interactions. Understanding how fast VOC blends recover, and whether recovery trajectories differ among bryophyte species and drought histories, are important next steps towards predicting bryophyte responses to future climate extremes.

## Supplementary Material

plag054_Supplementary_Data

## Data Availability

Data is available online at https://doi.org/10.6084/m9.figshare.30894017.
