# Supplementary material for: Altered volatile emissions and incomplete photosynthetic recovery after heatwave-associated drought and rewetting in the woolly feather-moss Tomentypnum nitens
Source: AoB Plants. 2026 Oct 15;18(5):plag054. doi: 10.1093/aobpla/plag054 (PMC13711661; doi:10.1093/aobpla/plag054)
Supplement: Supplementary file 1 [file plag054_supplementary_data.pdf]

Supplementary Information for:

Altered Volatile Emissions and Incomplete Photosynthetic Recovery after Heatwave-Associated Drought and Rewetting in the Woolly Feather-Moss *Tomentypnum nitens*

Supplementary table S1: List of 45 authentic standards used to quantify and identify VOCs.

Standard	Molecular Formula
isoprene	C5H8
2-butanone	C4H8O
hexane	C6H14
2-methylfuran	C5H6O
ethyl acetate	C4H8O2
toluene	C7H8
1-octene	C8H16
hexanal	C6H12O
2-furaldehyde	C5H4O2
trans-2-hexen-1-al	C6H10O
cis-3-hexen-1-ol	C6H12O
p-xylene	C8H10
o-xylene	C8H10
a-pinene	C10H16
camphene	C10H16
benzaldehyde	C7H6O
1-octen-3-ol	C8H16O
b-pinene	C10H16
myrcene	C10H16
octanal	C8H16O
cis-3-hexenyl acetate	C8H14O2
a-phellandrene	C10H16
3-carene	C10H16
p-cymene	C10H14
d-limonene	C10H16
benzyl alcohol	C7H8O
eucalyptol	C10H18O
ocimene	C10H16
y-terpinene	C10H16
acetophenone	C8H8O
terpinolene	C10H16
linalool	C10H18O
nonanal	C9H18O
(minus)-a-thujone	C10H16O
(plus)-camphor	C10H16O
(minus)-borneol	C10H18O
cis-3-hexenyl butyrate	C10H18O2
methyl salicylate	C8H8O3
bornyl acetate	C12H20O2
indole	C8H7N
(plus)-a-longipinene	C15H24
(minus)-isolongifolene	C15H24
b-caryophyllene	C15H24
a-humulene	C15H24
caryophyllene oxide	C15H24O

Supplementary table S2: Loading matrix table showing PCA loadings for all VOCs. Compound, shows the individual compounds ordered by the corresponding VOC group, MT (monoterpenes), SQT (sesquiterpenes), OVOC (oxygenated volatile organic compound), GLV (green leaf volatiles), HC (hydrocarbon) or OTHER (not belonging to any of the beforementioned groups). Class indicates the chemical class of the compound; MF lists the molecular formula.

Compound	Class	MF	PC1	PC2
**MT**				
**camphene**	MT	C10H16	0.08	-0.14
**a-phellandrene**	MT	C10H16	0.07	0.11
**unknown 8**	MT	C10H16	0.01	-0.06
**cyclohexene, 1-methyl-5-(1-**	MT	C10H16	-0.20	0.04
**ocimene**	MT	C10H16	0.18	0.07
**unknown 9**	oxygenated MT	C10H20O	0.16	0.11
**SQT**				
**(plus)-a-longipinene**	SQT	C15H24	0.16	-0.09
**unknown 12**	SQT	C15H24	-0.09	0.01
**b-caryophyllene**	SQT	C15H24	0.16	0.05
**unknown 15**	oxygenated SQT	C15H26O	0.03	0.10
**caryophyllene oxide**	oxygenated SQT	C15H24O	0.09	-0.02
**unknown 17**	oxygenated SQT	C15H24O	0.09	-0.04
**OVOC**				
**2-Propanol, 2-methyl-**	alcohol	C4H10O	0.13	0.13
**unknown 1**	ovoc	C4H8O	0.06	-0.19
**2-methylfuran**	furan	C5H6O	0.12	-0.17
**ethyl acetate**	ester	C4H8O2	-0.11	0.03
**methyl propionate**	ester	C4H8O2	0.18	0.10
**3-methoxybut-1-ene**	ether	C5H10O	0.12	-0.03
**unknown 3**	furan	C4H6O	0.09	-0.21
**2-butanone, 3-methyl-**	ketone	C5H10O	0.08	-0.21
**Furan, tetrahydro-2-methyl-**	ether	C5H10O	0.15	-0.05
**2-pentanone**	ketone	C5H10O	0.06	-0.20
**3-pentanone**	ketone	C5H10O	0.17	-0.13
**1,4-dioxane**	ether	C4H8O2	-0.02	-0.13
**unknown 4**	unknown	C6H10O	0.20	0.01
**unknown 5**	ketone	C6H12O	-0.01	0.13
**2-furaldehyde**	furan	C5H4O2	0.09	-0.09
**benzaldehyde**	oxygenated	C7H6O	-0.02	-0.12
**1-octen-3-ol**	alcohol	C8H16O	-0.06	-0.11
**2(3h)-furanone, 5-ethyldihydro-**	furan	C6H10O2	0.19	-0.04
**acetophenone**	oxygenated	C8H8O	-0.03	-0.11
**nonanal**	aldehyde	C9H18O	0.11	0.08
**benzaldehyde dimethyl acetal**	oxygenated	C9H12O2	0.16	0.08
**benzene, (1-methoxyethenyl)-**	oxygenated	C9H10O	-0.01	0.03
**2-Hydroxymandelic acid, ethyl ester, di-**	ovoc	C16H28O4Si2	-0.15	0.08
**ethanol, 2-phenoxy-**	ether	C8H10O2	0.10	0.00
**nonanal dimethyl acetal**	ether	C11H24O2	0.17	0.08
**undecanal**	aldehyde	C11H22O	0.10	-0.03
**benzophenone**	oxygenated	C13H10O	-0.01	-0.03

**GLV**				
**heptanal**	aldehyde	C7H14O	0.06	-0.20
**hexanal**	aldehyde	C6H12O	0.08	-0.11
**cis-3-hexen-1-ol**	alcohol	C6H12O	0.18	0.03
**HC**				
**pentane, 3-methyl-**	alkane	C6H14	0.05	0.19
**hexane**	alkane	C6H14	0.15	0.06
**unknown 2**	alkane	C6H10	0.07	-0.16
**benzene**	benzenoid	C6H6	-0.03	-0.17
**toluene**	benzenoid	C7H8	0.05	-0.21
**unknown 6**	hc	C7H8	0.08	-0.22
**1-octene**	alkene	C8H16	-0.05	-0.07
**unknown 7**	alkene	C8H16	-0.08	-0.14
**p-xylene**	benzenoid	C8H10	0.10	-0.18
**heptane, 2,2,4,6,6-pentamethyl-**	alkane	C12H26	0.15	0.12
**decane**	alkane	C10H22	0.16	0.12
**undecane, 2-methyl-**	alkane	C12H26	0.13	0.11
**dodecane**	alkane	C12H26	-0.07	0.14
**unknown 10**	benzenoid	C12H16	-0.12	0.05
**unknown 11**	alkane	C13H28	-0.04	0.14
**dodecane, 4-methyl-**	alkane	C13H28	-0.08	0.13
**dodecane, 2-methyl-**	alkane	C13H28	-0.13	-0.05
**unknown 13**	benzenoid	C12H10	-0.03	-0.06
**tetradecane**	alkane	C14H30	-0.04	0.08
**biphenyl**	benzenoid	C12H10	0.00	0.08
**unknown 14**	benzenoid	C13H12	0.02	0.14
**unknown 16**	alkane	C19H40	-0.11	0.03
**OTHER**				
**ethyl bromide**	halogenated	C2H5Br	0.13	0.13
**urea**	nitrogen-containing	CH4N2O	0.14	0.00
**trichloromethane**	halogenated	CHCl3	0.18	0.01
**isobutyronitrile**	nitrogen-containing	C4H7N	0.05	-0.19
**methane, dibromo-**	halogenated	CH2Br2	0.10	0.17
**disulfide, dimethyl**	sulfur-containing	C2H6S2	0.10	-0.10
**6-Azaestra-1,3,5(10),6,8-pentaen-17-one,**	nitrogen-containing	C18H19NO2	0.03	0.05
**ethane, 1,2-dibromo-**	halogenated	C2H4Br2	0.14	0.14
**dibromoacetaldehyde**	halogenated	C2H2Br2O	0.19	0.04
**methane, tribromo-**	halogenated	CHBr3	0.14	0.14
**4-cyanocyclohexene**	nitrogen-containing	C7H9N	0.13	-0.06
**benzothiazole**	nitrogen-containing	C7H5NS	0.16	0.05
**indole**	nitrogen-containing	C8H7N	0.10	-0.02
